# Safe Reduction of Nulliparous, Term, Singleton, Vertex Cesarean Delivery Rates Using Multidisciplinary Quality Improvement Efforts

**DOI:** 10.1097/og9.0000000000000157

**Published:** 2026-03-12

**Authors:** Chandler McGee, Celeste A. Green, Lauren Shubert, Adewunmi Babalola, Audra Timmins, Stephanie Gonzales-Hughes, Sharon Burks, Khanh Nguyen, Erin Gonzales, Sheena Glover, Tara Barrick, Courtney Thompson, Grace Achim, Kristin Thorp, Anne-Marie Savage, Matt Carroll, Christina Davidson

**Affiliations:** Department of Obstetrics and Gynecology, Baylor College of Medicine, Houston, Texas; and Department of Obstetrics and Gynecology, Texas Children's Hospital, Houston, Texas.

## Abstract

**OBJECTIVE:**

The rate of cesarean deliveries in nulliparous, term, singleton, vertex (NTSV) patients at our hospital has been about 30% since we began tracking this measure in 2016. Our aim was to evaluate the effect of quality improvement (QI) initiatives on this metric.

**METHODS:**

Our Level IV urban academic center formed a multidisciplinary workgroup in April 2023. We reviewed NTSV cesarean delivery rates stratified by indication, physician practice, and race and ethnicity, which were regularly presented to the department and practice leaders. Nurses audited oxytocin management for adherence to hospital protocol and introduced nonpharmacologic options to help patients cope with labor pain. Retrospective chart review of all NTSV cesarean deliveries from May to October 2023 determined whether the indication for cesarean delivery met American College of Obstetricians and Gynecologists (ACOG) criteria for failed induction of labor, active-phase arrest, second-stage arrest, and prelabor/scheduled cesarean delivery. Results were presented at departmental meetings, and each physician was sent a scorecard with their total NTSV cesarean delivery and adherence to ACOG criteria. A retrospective cohort study was conducted around implementation of the QI efforts to safely reduce the NTSV cesarean delivery rate.

**RESULTS:**

Overall and race-stratified NTSV cesarean delivery rates decreased over the time period of the hospital QI efforts. The overall preintervention rate was 31.0%, and this decreased significantly to 27.7% after intervention (*P*=.02). The rate for non-Hispanic White patients decreased significantly (27.7% vs 22.2%, *P*=.02), whereas non-Hispanic Black patients experienced a trend toward rate reduction from before to after intervention (38.3% vs 31.6%, *P*=.05). The rate of severe newborn complications was the same before and after intervention (0.5% vs 0.5%, *P*=.99). Chart review identified 298 NTSV cesarean deliveries, of which 13.8% did not meet ACOG criteria. Failed induction of labor was the most common indication (23.4%) that did not meet criteria across all racial and ethnic groups. Scheduled cesarean delivery rates not meeting ACOG criteria were highest for White and Hispanic patients, driven by cesarean delivery for estimated fetal weight lower than ACOG thresholds.

**CONCLUSION:**

Our hospital NTSV cesarean delivery rate decreased without an increase in severe newborn complication rates. Our chart audit allowed us to identify an opportunity to target specific QI interventions. Sharing disaggregated data and detailed chart review to identify hospital-specific drivers of nonmedically indicated cesarean delivery may be strategies to safely reduce NTSV cesarean delivery rates and drive QI initiatives.

## Abstract 2

Multidisciplinary quality improvement efforts that integrate data disaggregation with hospital-specific drivers can lead to reductions in nulliparous, term, singleton, vertex cesarean delivery rates and racial and ethnic disparities without compromising neonatal outcomes.

Currently, 32% of all births in the United States are delivered by cesarean delivery. This rate has increased from only 5% in the 1970s.^1^ The causes for increased rates of cesarean delivery are multifactorial, with a significant contributor being the increase in primary cesarean deliveries.^2^ The largest increases in primary cesarean delivery have been observed in populations at low risk, specifically nulliparous, term, singleton, vertex (NTSV) pregnancies.^3^ The literature further shows disparities in cesarean delivery rates by race and ethnicity, with higher rates in non-Hispanic Black and Hispanic patients compared with non-Hispanic White patients.^4^

In 2022, U.S. News and World Report launched its second annual Maternity Services Survey and invited hospitals that offer maternity care to self-report their maternity data through an online survey. Primary data collection from hospitals was necessary because quality data available from other sources such as the Centers for Medicare & Medicaid Services were insufficient on their own to evaluate the quality of maternal and neonatal care.^5^ That year, the survey expanded to include new questions and data points based on recommendations from hospitals, professional organizations, and expert advisors. The U.S. News and World Report evaluated how well hospitals performed in childbirth using structure, process, and outcome measures, to include NTSV cesarean delivery rates (as defined by the Joint Commission [JC]), severe newborn complication rates, breast-milk feeding rates, vaginal birth after cesarean delivery rates, episiotomy rates, whether a hospital meets new federal criteria for “birthing-friendly” practices, and whether a hospital tracked and reported outcomes for patients of different races and ethnicities.^6^ The U.S. News and World Report adopted benchmarks to evaluate and categorize hospital NTSV cesarean delivery rates, including the Healthy People 2020 and 2030 goals of 23.9% and 23.6%, respectively,^7,8^ and assigned zero points to the metric if greater than 30%, which is used by the JC to denote consistently low-performing hospitals.^9^ Our hospital voluntarily participated in the survey, submitting overall rates and rates by race and ethnicity for the 2020 calendar year. In submitting this data, our hospital recognized that our overall rate was high and that our disaggregated rates mirrored national trends, with non-Hispanic Black patients demonstrating the highest rates. This prompted our hospital to implement a series of quality improvement (QI) interventions aimed at safely reducing our institutional rate and disparities. Our objective was to determine the effect of these interventions on NTSV cesarean delivery rates and infant outcomes.

## METHODS

We report our study design, data analysis, and outcomes following SQUIRE (Standards for Quality Improvement Reporting Excellence) 2.^10^ Our hospital is a Level IV maternal and neonatal hospital located in an urban medical center in Houston, Texas. This designation is assigned by the Texas Department of State Health Services and indicates that our hospital provides the highest level of comprehensive care for pregnant and postpartum patients.^11^ With an annual delivery volume of more than 6,500 births, our hospital is staffed by both academic and private-practice obstetric physicians and certified nurse midwives and supports the training of 48 obstetrics and gynecology residents, one of the largest residency programs in the United States.

In response to our observed NTSV cesarean delivery rates and disparities during collection of our data for submission to U.S. News and World Report, our hospital formed a multidisciplinary Promoting Vaginal Birth work group in April 2023 to identify driving factors, root causes, and potential QI interventions to safely affect the rates. The work group included physicians, nursing leadership, staff labor nurses, quality and patient safety nurses, and data analysts and was led by the hospital chief quality officer for obstetrics and gynecology (physician lead) and the director of women's services (nurse lead). A project charter was developed, integrating the Institute for Healthcare Improvement's project charter^12^ with an Equity Impact Assessment tool^13^ given the known disparities in cesarean delivery rates (Appendix 1, available online at http://links.lww.com/AOG/E562). The Institute for Healthcare Improvement charter is designed to answer the three essential questions of the Model for Improvement: 1) What are we trying to accomplish; 2) How will we know that a change is an improvement; and 3) What change can we make that will result in an improvement?^12^ Within the framework of the Institute for Healthcare Improvement charter, we embedded a racial equity impact assessment to systematically examine how different racial and ethnic groups would likely be affected by our proposed actions or decisions given the known racial disparities in our NTSV cesarean delivery rates. Our project aim, using a SMARTIE (specific, measurable, attainable, relevant, time-based, inclusive, and equitable) framework,^14^ was to increase adherence to national guideline criteria for NTSV cesarean delivery indications and to reduce the rate of nonmedically indicated NTSV cesarean deliveries by 80% and the non-Hispanic Black to non-Hispanic White and non-Hispanic Asian to non-Hispanic White disparity by 50% by December 31, 2024, through implementation of promoting vaginal birth interventions (Appendix 1, http://links.lww.com/AOG/E562).

Given the potential contribution of the coronavirus disease 2019 (COVID-19) pandemic to the 2020 hospital NTSV cesarean delivery rates that were submitted to the U.S. News and World Report survey, the hospital quality team did a historical and more contemporary review of NTSV cesarean delivery rates, overall and by race and ethnicity. In addition to racial and ethnic disaggregation, rates were stratified by prenatal care practice group to identify practice patterns that may affect the rates. Rates were reviewed from January 1, 2016, to May 31, 2023.

Through an adaption of existing bundles promoting vaginal birth^15,16^ and consultation with the Illinois Perinatal Quality Collaborative, which has extensive expertise and resources in reducing primary cesareans,^17^ the work group developed a series of planned interventions to promote safe vaginal birth that were rooted in the findings from our disaggregated data analysis.

PDSA (plan-do-study-act) Cycle 1 involved data sharing by the quality and safety team. The chief quality officer presented the hospital's NTSV rates overall, stratified by race and ethnicity, and stratified by clinical practice (deidentified) at monthly hospital department meetings, beginning in September 2023. In addition, the chief quality officer met individually with all clinical practice leaders in September 2023 to unblind their practice-specific rates and to discuss clinical practice patterns that may be contributing to the rates. In addition to NTSV cesarean delivery rates, vaginal birth after cesarean delivery and severe newborn complication rates were shared with practice leaders to serve as balancing measures. Severe newborn complication is a JC composite measure of severe unexpected complications among full-term newborns with no preexisting conditions and includes neonatal death, transfer to another hospital for a higher level of care, severe birth injuries such as intracranial hemorrhage or nerve injury, neurologic damage, severe respiratory, and infectious complications such as sepsis.^18^

PDSA Cycle 2 involved a nursing audit of oxytocin administration practices to determine adherence to the hospital policy. Audits were conducted on all patients who underwent induction of labor between January 1, 2022, and May 31, 2022, to have a historical comparison exclusive of pandemic rates and limited to contemporary labor practices. These audits were coupled with an anonymous oxytocin use assessment survey sent out to nursing staff to help identify gaps in practices and contributing factors to nonadherence to the oxytocin checklist. The survey, consisting of four open-ended questions, was distributed to staff nurses in February 2024 and remained open for 1 month. Nursing champions from the Promoting Vaginal Birth work group served as resources on labor and delivery for questions about oxytocin titration.

PDSA Cycle 3 involved a chart audit of all NTSV cesarean deliveries that were performed between May 1, 2023, and October 31, 2023, to explore postpandemic contemporary labor management. Indications for NTSV cesarean delivery were grouped into the following categories: failed induction of labor, active-phase arrest, second-stage arrest, scheduled cesarean delivery, nonreassuring fetal heart rate (FHR), and maternal request. Chart reviews were conducted by an obstetrics and gynecology resident, a maternal–fetal medicine fellow, an attending obstetrician, the chief quality officer, and a labor nurse. Each chart was reviewed by at least one physician and one nurse. The charts were audited for indication for cesarean delivery and if clinical criteria for cesarean delivery were met for labor dystocia based on the American College of Obstetricians and Gynecologists (ACOG) and the Society for Maternal Fetal Medicine (SMFM) Obstetric Care Consensus,^19^ which was replaced by an ACOG Clinical Practice Guideline during the course of chart audits.^20^ The ACOG publications were also used to determine adherence to recommendations regarding cesarean delivery for fetal macrosomia and cesarean delivery on maternal request.^21,22^ Charts were deemed to meet ACOG criteria for cesarean delivery if the following were met:

- Failed induction of labor: 12 hours or more of oxytocin after rupture of membranes; if oxytocin was paused, total duration administered of 12 hours or more
- Active-phase arrest: No cervical change for 6 hours or more with inadequate contractions (Montevideo units less than 200) or 4 hours or more with adequate contractions
- Second-stage arrest: 3 or more hours pushing without epidural or 4 or more hours in nulliparous patients
- Nonreassuring FHR: Accepted if documentation supported attempted intrauterine resuscitation
- Scheduled cesarean delivery for macrosomia: estimated fetal weight exceeding 4500 g (with diabetes mellitus, regardless of type of diabetes or use and type of medication) or exceeding 5000 g (without diabetes)
- Cesarean delivery on maternal request: Chart documentation supported maternal request and physician documentation of counseling of risks and benefits

If there was any uncertainty from a chart reviewer on whether the indications for cesarean delivery met ACOG criteria, they were sent to the chief quality officer for review and final determination. Aggregated results were shared at the June 2024 department meeting, and physicians were emailed individualized scorecards from the chief quality officer (Appendix 2, http://links.lww.com/AOG/E562) in July 2024, with a detailed overview of their chart audit assessment on request.

Findings from the chart audit formed the basis for the subsequent PDSA cycles. Cycle 4 involved development of several tools and interventions from the workgroup nurses, including a patient education infographic about induction of labor in English and Spanish (Appendix 3, http://links.lww.com/AOG/E562) that was adapted from a tool shared by colleagues at the University of Pennsylvania Obstetrics and Gynecology Department. Based on feedback the chief quality officer received after sharing findings from the chart audits with patient representatives from our hospital's Patient Advisory Council, this infographic was emailed to each patient scheduled for induction of labor and available in every labor and delivery room as a visual aid on admission for delivery. To offer a variety of nonpharmacologic techniques to cope with labor pain,^23^ a “comfort cart” was designed and available to all women in labor with an accompanying visual aid in English and Spanish (Appendix 4, http://links.lww.com/AOG/E562). These tools were coupled with nursing peer education from the work group champions around physiologic labor support. Cycle 5 involved an update to the hospital guideline on timing of delivery and management of labor to include checklists and algorithms for intrapartum management and evidence-based criteria for cesarean delivery^20^ as well as a standardized note template to document the indication for cesarean delivery (Appendix 5, http://links.lww.com/AOG/E562).

Throughout the PDSA cycles, NTSV cesarean delivery rates—overall and by race and ethnicity—were regularly presented at hospital department and quality and patient safety meetings, along with associated interventions. We then conducted a retrospective cohort study around implementation of the QI efforts to safely reduce the NTSV cesarean delivery rate. Our postintervention cohort included those NTSV cesarean deliveries that occurred after the first targeted intervention from the Promoting Vaginal Birth work group, which was data sharing in September 2023; our preintervention cohort included NTSV cesarean deliveries performed between January 1, 2022, and August 31, 2023, to have a historical comparison exclusive of pandemic rates and limited to contemporary labor practices. Our primary outcome was overall NTSV cesarean delivery rate. Secondary outcomes included NTSV cesarean delivery rate for each racial and ethnic group, the rate of change for each group between the intervention time periods, and a balancing metric of severe newborn complications. For race and ethnicity classification, our hospital admissions team collects self-reported race and ethnicity data on all admitted patients and uses the Office of Management and Budget standards,^24^ with ethnicity being either Hispanic/Latina or non-Hispanic/Latina and race being categorized as American Indian or Alaska Native, Asian, Black or African American, White, Native Hawaiian, or Other Pacific Islander. For purposes of reporting our hospital outcomes, we adopted the following categories: non-Hispanic Black, non-Hispanic White, Hispanic, non-Hispanic Asian, and none of the above. The χ^2^ test was used for categorical outcomes. Given the iterative QI nature of the study, data were displayed in run charts (Minitab 17.3.1) to assess for trends over time, and potential confounding was not assessed. Values of *P*<.05 were considered statistically significant. This study was approved by the Baylor College of Medicine IRB for Human Subject Research.

## RESULTS

During the study period, there were 16,527 total deliveries: 11,100 before intervention and 5,427 after intervention. There were no differences in patient age, race and ethnicity, body mass index (BMI), gestational age at delivery, and payer status between the two groups (Table 1).

**Table 1.** Maternal Characteristics of All Deliveries

Maternal Characteristics			
Characteristic	Preintervention Baseline (N=11,100)	After Intervention (N=5,427)	*P*
Age, mean±SD (y)	30.5±5.8	30.6±5.8	.28
Delivery gestational age, median (IQR) (wk)	39.0 (2.3)	38.9 (2.1)	.84
BMI at delivery, median (IQR)	32.0 (8.8)	31.7 (8.7)	.49
Race and ethnicity			
Hispanic	4,567 (41.1)	2,305 (42.5)	.10
Non-Hispanic Asian	713 (6.4)	346 (6.4)	.91
Non-Hispanic Black	2,362 (21.3)	1,094 (20.2)	.10
Non-Hispanic White	3,302 (29.7)	1,593 (29.3)	.60
None of the above	156 (1.4)	89 (1.6)	.24
Delivery mode			
Cesarean	4,360 (39.3)	2,031 (37.4)	.02
Vaginal	6,740 (60.7)	3,396 (62.6)	.02
Insurance			
Commercial	6,854 (61.7)	3,360 (61.9)	.84
Medicaid	4,170 (37.6)	1997 (36.8)	.34

IQR, interquartile range; BMI, body mass index.

Values are n (%) unless indicated otherwise.

This table represents the maternal characteristics from all deliveries during the study time period. The preintervention phase is from January 1, 2022, to August 31, 2023; the postintervention time period is from September 1, 2023, to June 30, 2024.

**Fig. 1.**
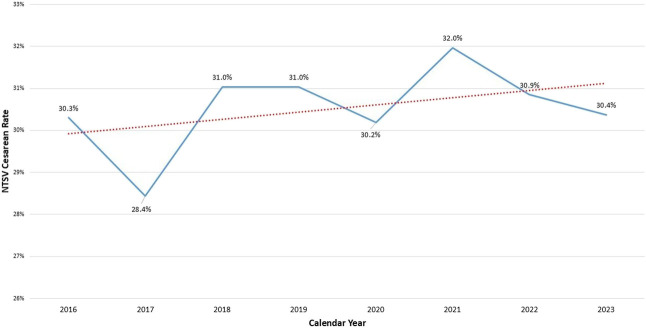
Preintervention annual nulliparous, term, singleton, vertex (NTSV) cesarean delivery rate, overall, from January 1, 2016, to May 31, 2023.

**Fig. 2.**
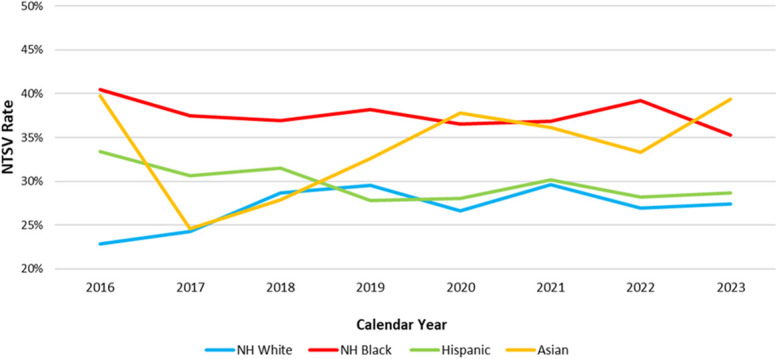
Preintervention annual nulliparous, term, singleton, vertex (NTSV) cesarean delivery rate, by race and ethnicity, from January 1, 2016, to May 31, 2023. NH, Non-Hispanic.

Data from PDSA Cycle 1 demonstrated that prenatal practice group-specific NTSV cesarean delivery rates were highest for the maternal–fetal medicine practice (average rate 43.0% between January 1, 2019, and May 31, 2023) and lowest for the two practices that had a physician assigned to labor and delivery and physically present in the hospital at all times (average rate 26.0% between January 1, 2019, and May 31, 2023). The other practices all used a model of labor and delivery coverage while also seeing patients in the ambulatory setting and overnight labor and delivery call coverage from home; their average rate was 32.0% (blinded data presented in Appendix 1, http://links.lww.com/AOG/E562). Given the higher rate of NTSV cesarean deliveries in the MFM practice, PDSA Cycle 1 also included a comparison of rates using the JC definition of the metric, which excludes only placenta previa from the numerator, and the SMFM definition of low-risk cesarean deliveries, which additionally excludes some high-risk conditions such as human immunodeficiency virus (HIV), herpes simplex virus, eclampsia, vasa previa, history of uterine surgery, maternal cardiovascular disease, and some fetal anomalies.^25^ When we reviewed rates in more than 9,000 NTSV cesarean deliveries between 2018 and 2023 at our hospital, the rates obtained from the JC metric and the SMFM metric were nearly identical, with the primary indication for cesarean delivery being FHR concern in both metric definitions (data not shown). A more longitudinal review of our hospital NTSV cesarean delivery rates, overall (Fig. 1) and stratified by race and ethnicity (Fig. 2), demonstrated that the 2020 rates submitted to U.S. News and World Report could not be attributed solely to practice changes during the COVID-19 pandemic. Rather, they reflected a pattern of racial disparities and overall rates exceeding 30%.

*McGee. Safe Reduction of Low-Risk Cesareans. O&G Open 2026*.

*McGee. Safe Reduction of Low-Risk Cesareans. O&G Open 2026*.

Oxytocin audits of 365 patients who underwent induction of labor, as described in PDSA Cycle 2, demonstrated a baseline interval ranging from 32 to 54 minutes between dosing increases. Forty-two staff nurses submitted responses to the survey and indicated that common barriers to more timely increases included pain management and delays from administration of neuraxial analgesia. Survey responses also indicated that some nurses would turn oxytocin off if the patient was experiencing severe pain while awaiting neuraxial placement and that they would request a pause in the oxytocin administration if the patient had been receiving it for more than 12 hours and the patient was not making adequate cervical change. A median dosing interval of 30 minutes was achieved after nursing education, comfort carts, and oxytocin champions were embedded on labor and delivery.

**Fig. 3.**
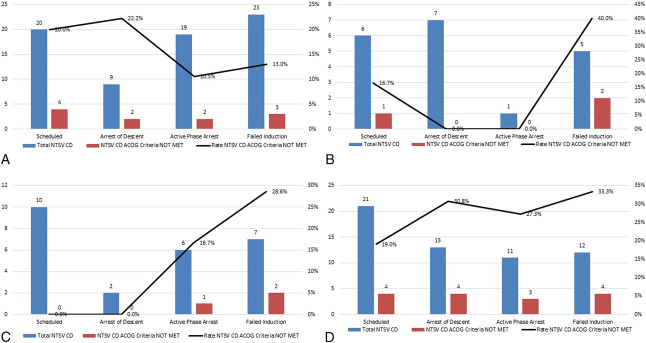
Indication for nulliparous, term, singleton, vertex (NTSV) cesarean delivery (CD) and adherence to American College of Obstetricians and Gynecologists (ACOG) criteria, stratified by race and ethnicity. Hispanic (**A**), non-Hispanic Asian (**B**), non-Hispanic Black (**C**), and non-Hispanic White (**D**).

Chart audits described in PDSA Cycle 3 identified 298 NTSV cesarean deliveries that were reviewed: 47 (15.8%) were performed for failed induction of labor, 38 (12.8%) for active-phase arrest, and 31 (10.4%) for second-stage arrest; 58 (19.5%) were scheduled/before labor, 110 (36.9%) for FHR concern, and 14 (4.7%) for other reasons. Among these, 41 (13.8%) did not meet ACOG/SMFM criteria for cesarean delivery based on chart documentation. The most common indication that did not meet criteria was failed induction of labor, accounting for 23.4% of nonadherent cases across all racial and ethnic groups; 19.4% did not meet criteria for second-stage arrest, 15.8% for active-phase arrest, 15.5% for scheduled delivery, and 0.9% for FHR concerns. Prelabor/scheduled cesarean deliveries that did not meet ACOG thresholds were most frequently observed in non-Hispanic White and Hispanic patients (Fig. 3). The most common indications for scheduled cesarean deliveries were prior nonobstetric uterine surgery (eg, myomectomy) and fetal macrosomia, with suspected macrosomia being the indication associated with the highest rate of nonadherence to ACOG criteria because of a documented estimated fetal weight that was below the ACOG-defined thresholds for surgical delivery. During the chart audit time period, the overall NTSV cesarean delivery rate was 30.1%; if the 41 cases that did not meet ACOG criteria had all resulted in vaginal deliveries, the chart audit rate would have decreased to 25.9%.

*McGee. Safe Reduction of Low-Risk Cesareans. O&G Open 2026*.

**Fig. 4.**
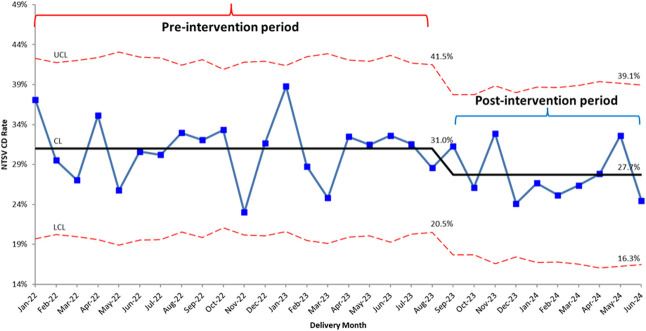
Overall monthly nulliparous, term, singleton, vertex (NTSV) cesarean delivery (CD) rate during the preintervention and postintervention time periods. UCL, upper control limit; LCL, lower control limit.

Overall and race-stratified NTSV cesarean delivery rates decreased over the time period of the hospital QI efforts (Table 2 and Fig. 4). The overall preintervention rate was 31.0%, which decreased significantly to 27.7% after intervention (*P*=.02). The rate for non-Hispanic White patients decreased significantly (27.7% vs 22.2%, *P*=.02), whereas non-Hispanic Black patients experienced a trend toward rate reduction from before to after intervention (38.3% vs 31.6%, *P*=.05). The rates for non-Hispanic Asian and Hispanic patients were unchanged. The hospital reached its lowest calendar year rate of 27.2% by June 2024. The severe newborn complication rate during the postintervention time period was unchanged compared with before intervention (0.5% vs 0.5%, *P*=1.0). Because of the departure of two clinical practices from our hospital in June 2024—the midwifery practice and the practice with the lowest NTSV cesarean delivery rates—our postintervention time period was terminated at June 2024 to compare similar patient populations before and after intervention. The lowest monthly NTSV cesarean delivery rates our hospital have experienced, however, were 25.8% in August 2024 and 21.5% in September 2024, which were the 2 months immediately after the physician scorecard distribution from PDSA Cycle 3.

**Table 2.** Nulliparous, Term, Singleton, Vertex Cesarean Delivery Rate Before and After Intervention, Overall and by Race and Ethnicity

	Preintervention NTSV CD, n	Postintervention NTSV CD, n	% Change	*P*
Hispanic	352 (28.8)	170 (28.1)	2.4	.76
Non-Hispanic Asian	82 (34.3)	44 (37.6)	9.6	.54
Non-Hispanic Black	241 (38.3)	99 (31.6)	17.5	.05
Non-Hispanic White	284 (27.7)	105 (22.1)	20.2	.02
Overall	982 (31.0)	425 (27.7)	10.6	.02

NTSV, nulliparous, term, singleton, vertex; CD, cesarean delivery.

This table represents the NTSV cesarean delivery rate overall and for each racial and ethnic group in the preintervention phase compared with the postintervention phase. The preintervention phase is from January 1, 2016, to March 30, 2023; the postintervention time period is from April 1, 2023, to June 30, 2024.

## DISCUSSION

Our findings demonstrate that focused, multidisciplinary QI initiatives can lead to a meaningful reduction in NTSV cesarean delivery rates without increasing neonatal morbidity. By combining department-wide education, structured audits, and feedback through individualized scorecards, we were able to encourage adherence to established national guidelines. In addition, our QI approaches align with those recently recommended by ACOG as strategies for the safe reduction of primary cesarean deliveries.^26^

The identification of nonadherent cesarean deliveries—particularly those labeled as failed induction of labor and prelabor/scheduled cesarean deliveries—provided targets for further intervention. Failed induction of labor emerged as the most frequent indication that did not meet ACOG/SMFM criteria, with chart audits revealing that some cesarean deliveries were secondary to patient request attributed to a perception that the process was taking too long. This, coupled with the nursing survey responses regarding barriers to timely oxytocin titration, formed the basis for targeted patient and clinician educational tools. Similarly, scheduled cesarean deliveries for macrosomia in non-Hispanic White and Hispanic patients suggested a need for additional physician education regarding evidence-based thresholds to recommend abdominal delivery. It is thus not surprising that the most significant rate reduction from these targeted efforts was observed in non-Hispanic White patients because they had the highest rates of cesarean delivery not meeting ACOG criteria from the chart audits performed during PDSA Cycle 3. The severe newborn complication rate remained stable after enactment of the interventions, supporting the neonatal safety of our strategies aimed at reducing primary cesarean deliveries.

Our study emphasizes the advantages of stratifying data in that disaggregated analysis allowed a better understanding of the largest, more specific drivers behind NTSV cesarean delivery rates and facilitated identification and implementation of targeted QI interventions. Our study also demonstrates the utility in consulting with other organizations involved in similar QI initiatives to adopt and adapt processes and tools that have already been developed and implemented, as demonstrated by our partnerships with the Illinois Perinatal Quality Collaborative and the University of Pennsylvania. Although many resources may exist for QI interventions, implementation and uptake can be enhanced when sharing practices that work well and barriers to success across organizations.

Our study is limited by the lack of follow-up chart audits to determine whether the rate of adherence to ACOG/SMFM criteria improved with the targeted QI interventions. We additionally did not target interventions around FHR interpretation and management, thus limiting our ability to have a more significant effect on NTSV cesarean delivery rates because FHR concerns are the primary indication for NTSV cesarean delivery in our patients. We intentionally chose to focus our initial efforts on labor dystocia because there are well-defined criteria for labor abnormalities that justify cesarean delivery. We are also limited by our fixed postintervention time period. Because of the departure of two clinical practices from our hospital in June 2024, additional data would not be representative of the same patient population to whom the interventions were applied. We further acknowledge that although we were able to achieve a statistically significant reduction in our NTSV cesarean delivery rate, it is still higher than the national goal. Although we have targeted our interventions around opportunities to safely reduce our NTSV cesarean delivery rate, we have not yet established a goal rate for our patient population.

In conclusion, through an organized multidisciplinary approach, our institution achieved a meaningful reduction in the NTSV cesarean delivery rate without a subsequent increase in neonatal complications. Future efforts will focus on continued audit and feedback cycles, particularly of prelabor/scheduled NTSV cesarean deliveries and cesarean deliveries for FHR concerns, and strategies to reduce nonindicated cesareans. Sharing disaggregated data and identifying hospital-specific drivers are essential components of safe, effective cesarean reduction strategies.
